# Supplementary material for: Home-Based Measurements of Dystonia in Cerebral Palsy Using Smartphone-Coupled Inertial Sensor Technology and Machine Learning: A Proof-of-Concept Study
Source: Sensors (Basel). 2022 Jun 9;22(12):4386. doi: 10.3390/s22124386 (PMC9231145; doi:10.3390/s22124386)
Supplement: Supplementary file 1 [file sensors-22-04386-s001.zip › sensors-1733101-supplementary.pdf]

Supplementary S2: Samples per clinical score for each subject.

Dataset	Number of videos	Dystonia lower extremity samples	Dystonia upper extremity samples
**Subject 1**	**29**	**Total: 720**	**Total: 726**
		Score 0: 710	Score 0: 188
		Score 1: 9	Score 1: 117
		Score 2: 1	Score 2: 231
		Score 3: 0	Score 3: 164
		Score 4: 0	Score 4: 26
**Subject 2**	**14**	**Total: 189**	**Total: 186**
		Score 0: 71	Score 0: 40
		Score 1: 64	Score 1: 74
		Score 2: 48	Score 2: 63
		Score 3: 6	Score 3: 8
		Score 4: 0	Score 4: 0
**Subject 3**	**1**	**Total: 24**	**Total: 24**
		Score 0: 19	Score 0: 1
		Score 1: 5	Score 1: 11
		Score 2: 0	Score 2: 11
		Score 3: 0	Score 3: 1
		Score 4: 0	Score 4: 0
**Subject 4**	**7**	**Total: 120**	**Total: 125**
		Score 0: 86	Score 0: 85
		Score 1: 2	Score 1: 38
		Score 2: 32	Score 2: 2
		Score 3: 0	Score 3: 0
		Score 4: 0	Score 4: 0
**Subject 5**	**12**	**Total: 388**	**Total: 441**
		Score 0: 385	Score 0: 222
		Score 1: 3	Score 1: 209
		Score 2: 0	Score 2: 10
		Score 3: 0	Score 3: 0
		Score 4: 0	Score 4: 0
**Subject 6**	**4**	**Total: 66**	**Total: 66**
		Score 0: 66	Score 0: 0
		Score 1: 0	Score 1: 8
		Score 2: 0	Score 2: 50
		Score 3: 0	Score 3: 8
		Score 4: 0	Score 4: 0
**Subject 7**	**17**	**Total: 334**	**Total: 336**
		Score 0: 99	Score 0: 0
		Score 1: 157	Score 1: 0
		Score 2: 54	Score 2: 246
		Score 3: 24	Score 3: 90
		Score 4: 0	Score 4: 0
**Subject 8**	**14**	**Total: 336**	**Total: 298**
		Score 0: 272	Score 0: 75
		Score 1: 64	Score 1: 165
		Score 2: 0	Score 2: 56
		Score 3: 0	Score 3: 2
		Score 4: 0	Score 4: 0

**Subject 9**	**14**	**Total: 588**	**Total: 583**
		Score 0: 162	Score 0: 2
		Score 1: 247	Score 1: 163
		Score 2: 167	Score 2: 390
		Score 3: 12	Score 3: 28
		Score 4: 0	Score 4: 0
**Subject 10**	**10**	**Total: 514**	**Total: 510**
		Score 0: 514	Score 0: 54
		Score 1: 0	Score 1: 359
		Score 2: 0	Score 2: 86
		Score 3: 0	Score 3: 11
		Score 4: 0	Score 4: 0
**Subject 11**	**6**	**Total: 478**	**Total: 444**
		Score 0: 0	Score 0: 0
		Score 1: 68	Score 1: 50
		Score 2: 373	Score 2: 218
		Score 3: 37	Score 3: 176
		Score 4: 0	Score 4: 0
**Subject 12**	**20**	**Total: 774**	**Total: 1242**
		Score 0: 31	Score 0: 11
		Score 1: 528	Score 1: 570
		Score 2: 209	Score 2: 505
		Score 3: 6	Score 3: 154
		Score 4: 0	Score 4: 2
**Generalized**	**148**	**Total: 4533**	**Total: 4976**
		Score 0: 2417	Score 0: 672
		Score 1: 1174	Score 1: 1774
		Score 2: 860	Score 2: 1984
		Score 3: 82	Score 3: 519
		Score 4: 0	Score 4: 27
